# Case Report: Conventional therapy versus volanesorsen in two sisters with familial chylomicronemia syndrome

**DOI:** 10.3389/fnut.2026.1749264

**Published:** 2026-02-09

**Authors:** Javier Vega, Benjamín Torres, Alberto Maiz, José L. Santos

**Affiliations:** 1 Department of Nutrition, Diabetes and Metabolism. School of Medicine, Pontificia Universidad Católica de Chile, Santiago, Chile; 2 Department of Health Sciences and Institute for Sustainability and Food Chain Innovation (IS-FOOD), Universidad Publica de Navarra, Pamplona, Spain; 3 School of Medicine, Pontificia Universidad Católica de Chile, Santiago, Chile

**Keywords:** apolipoprotein C-III, case-report, familial chylomicronemia syndrome, pancreatitis, triglycerides, volanesorsen

## Abstract

Familial chylomicronemia syndrome (FCS) is a rare autosomal recessive disorder characterized by severe hypertriglyceridemia and caused by mutations in genes involved in chylomicron metabolism. Dietary management includes a very-low-fat diet, restriction of simple carbohydrates and alcohol, supplementation with medium-chain triglycerides, essential fatty acids, and fat-soluble vitamins; however, long-term adherence is often poor and nutritional therapy alone is insufficient. We report two adult Chilean sisters with FCS caused by the homozygous Q97X mutation in the APOA5 gene. Both patients experienced severe hypertriglyceridemia (>5,000 mg/dL) and recurrent episodes of acute pancreatitis. One sister was treated with volanesorsen, an antisense oligonucleotide, receiving a weekly dose of 285 mg, which was repeated every 3 weeks due to thrombocytopenia. When combined with structured nutritional counseling, pharmacological treatment achieved a marked reduction in plasma triglycerides to <250 mg/dL and a substantial improvement in quality of life. The other sister was managed with conventional therapy due to a lack of health insurance coverage for volanesorsen. She presented persistent hypertriglyceridemia and recurrent hospitalizations, underscoring the challenges of access to advanced therapies in limited-resource settings. While volanesorsen offers a promising therapeutic alternative, equitable access remains a critical issue, particularly in health systems of low-to middle-income regions.

## 1 Introduction

Familial chylomicronemia syndrome (FCS; OMIM # 238600), also known as type I hyperlipoproteinemia according to the Fredrickson classification (1), is a rare autosomal recessive disease caused by biallelic mutations in genes involved in triglyceride-rich lipoprotein metabolism that causes defective clearance of triglycerides from circulating chylomicrons. It is characterized by chylomicronemia and consequently severe hypertriglyceridemia, over 10 to 100 times the normal value in asymptomatic patients (2), with a notable increased risk of acute pancreatitis (3). Patients with FCS usually show lipemic plasma that can be accompanied by eruptive xanthomas, lipemia retinalis, and neurological manifestations such as memory loss, irritability, cognitive dysfunction, and decreased quality of life (4, 5). It has been reported that around 80% of FCS cases carry biallelic mutations in the LPL gene encoding the lipoprotein lipase protein (6, 7). The rest of the patients carry mutations in other genes encoding proteins that participate in chylomicron metabolism and LPL function (APOC2, GPIHBP1, APOA5, or LMF1) (8).

A strict very low-fat diet is a key component of the nutritional management of FCS (9). The difficulty in adhering to a strict low-fat diet and the lack of effect of standard pharmacological approaches used in mild hypertriglyceridemia boosted the research on novel pharmacological treatments for FCS (2). After the discovery of a loss-of-function mutation in the APOC3 gene causing a cardioprotective plasma lipid profile (10), intense research led to the development of therapies targeting the inhibition of the apolipoprotein C-III protein (ApoC-III) (11). Volanesorsen is an antisense oligonucleotide that inhibits hepatic apolipoprotein C-III synthesis and constitutes a new therapeutic alternative for managing FCS patients (12, 13). Few cases of FCS have been reported in Chile and Latin America (14, 15). This study aims to show the clinical report of two Chilean sisters affected by FCS carrying the homozygous loss-of-function mutation Q97X in the APO5 gene (14). The high cost of the volanesorsen therapy and the features of the Chilean health system led to a situation in which only one of the two affected sisters with FCS received volanesorsen treatment, while the other one did not receive such treatment.

## 2 Case report

### 2.1 Family history

A four-generation extended family tree, including consanguineous relationships, has been previously published (14). The father of both index cases 1 and 2 (see below) was affected with type 2 diabetes, dyslipidemia, and acute fatal myocardial infarction at age 69. The mother was diagnosed with type 2 diabetes, dyslipidemia, and atherosclerotic vascular disease. The carrier status of the APOA5 Q97X (the same homozygotic gene mutation found in the FCS sisters reported herein) was also confirmed in the mother and several relatives.

### 2.2 Case 1

Female adult with a history of dyslipidemia since early childhood, previously of normal weight, and with no other relevant medical history. At age 25, she presented with an episode of acute pancreatitis during her first pregnancy, with plasma triglyceride levels reaching 3,000 mg/dL. Initial management included fibrates, niacin, and omega-3 fatty acids, along with modifications in her diet. During her second pregnancy, plasmapheresis was required due to triglyceride levels greater than 5,000 mg/dL. At age 40, she began a new treatment with fibrates, acipimox, and pravastatin, but experienced an increase in plasma creatine kinase up to 9,248 mg/dL, without presenting renal failure. Due to this increase, treatment was discontinued, but plasma triglyceride levels rose again to 5,500 mg/dL. Despite the good adherence to a low-fat diet, her plasma triglycerides remained above 500 mg/dL. At age 53, due to persistent hypertriglyceridemia and a family history of a sister with a similar diagnosis, a genetic study was performed that revealed homozygosity for the nonsense mutation Q97X of the APOA5 gene, responsible for her severe dyslipidemia. Until that time, she had not developed diabetes, arterial hypertension, or hypothyroidism.

**Figure 1:**
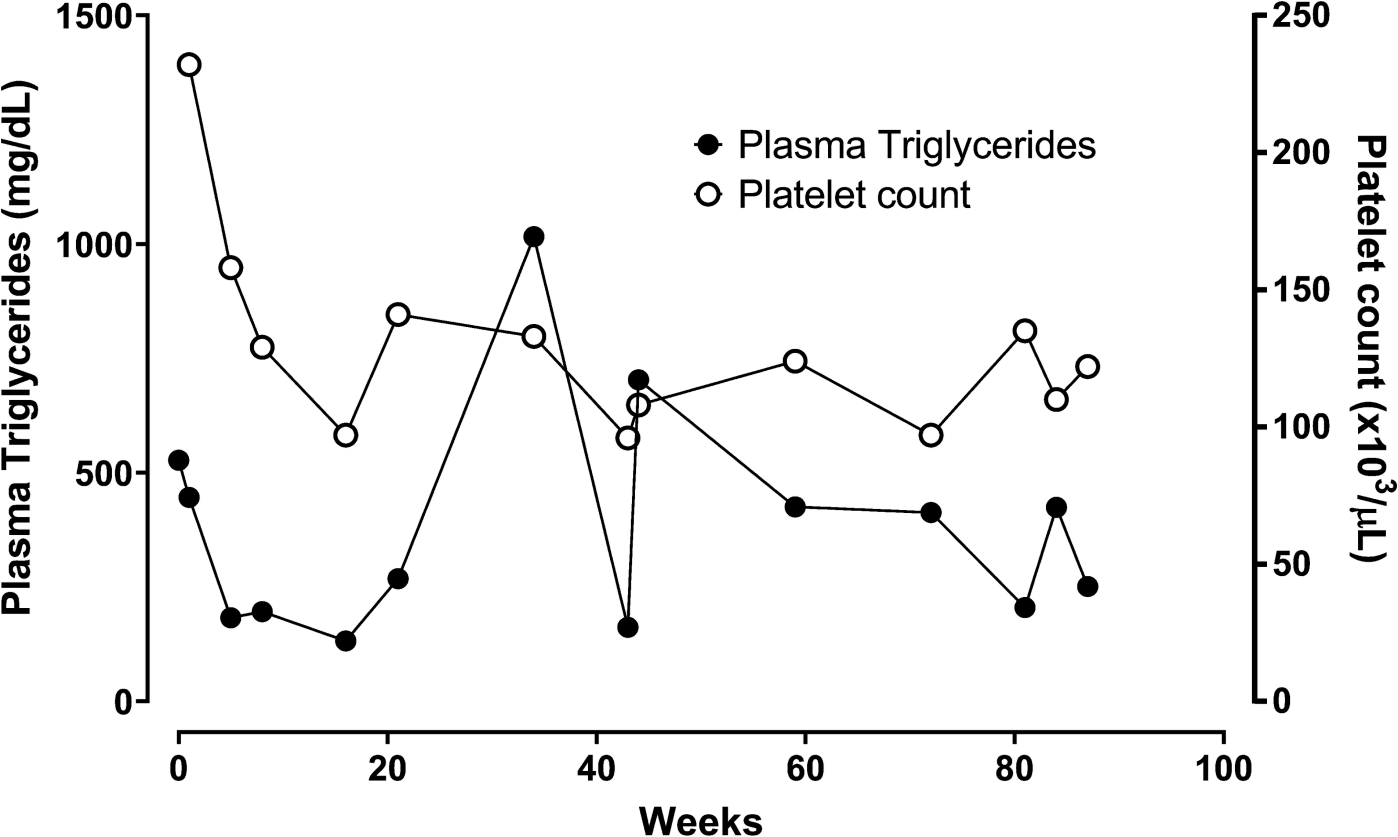
Weekly changes in triglyceride levels and platelets from the start of treatment with volanesorsen affected with FCS (case 1).

Current treatment: At the age of 59 years, the patient began treatment with volanesorsen at a dose of 285 mg/week subcutaneously. Since the beginning of this treatment, her plasma triglyceride levels have remained below 250 mg/dL (Figure 1). She has experienced local allergic reactions with erythema and mild pruritus at the puncture site. After 3 months of treatment, the administration was adjusted to every 2 weeks. At week 16, the treatment was discontinued temporarily because the patient developed thrombocytopenia (97,000/μL). Over a follow-up period of 90 weeks, with dose of volanesorsen adjustments to 285 mg every 3 weeks, the patient has maintained plasma triglyceride levels ranging from 161 to 705 mg/dL. During this period, the patient required temporary suspension of treatment on three occasions due to platelet counts falling below 100,000/μL. Since starting treatment, she has been asymptomatic and managed adequately with dietary restriction schemes.

### 2.3 Case 2

Female adult case with a history of dyslipidemia since early childhood. At age 30, she developed acute pancreatitis during her second pregnancy, with triglyceride levels above 9,000 mg/dL. Since then, she has been treated with a low-fat diet, avoiding refined carbohydrates and alcohol, and multiple medications, including fenofibrate, gemfibrozil, ciprofibrate, acipimox, orlistat, simvastatin, ezetimibe, niacin, and omega-3 fatty acids. In the last 10 years, plasma triglyceride levels have varied between 2,527 and 10,400 mg/dL, causing eruptive xanthomas, lipemia retinalis, and paresthesia. The patient required several hospitalizations due to abdominal pain and episodes of hypertriglyceridemia greater than 10,000 mg/dL, but without pancreatitis, where plasmapheresis has been performed, resulting in a good initial response but subsequent rise in plasma triglycerides. At 52 years of age, a genetic study was conducted that confirmed homozygosity for the nonsense mutation Q97X of the APOA5 gene, which explains her severe dyslipidemia. At 54 years of age, she was diagnosed with type 2 diabetes and began treatment with metformin and saxagliptin, followed by insulin in a basal-bolus regimen, with a glycated hemoglobin level near 6.7%. Current Treatment: The patient continues on a strict low-fat diet and multiple lipid-lowering and glucose-lowering therapies. Despite adherence to nutritional and pharmacological treatment, plasma triglyceride levels have remained poorly controlled, fluctuating between 510 and 6,360 mg/dL (mean 5,177 ± 924 mg/dL), with ongoing disease burden and recurrent healthcare utilization.

## 3 Discussion

Patients with FCS are at increased risk of developing acute pancreatitis (3). This risk increases by 4% for every 100 mg/dL above 1,000 mg/dL (16). The treatment, therefore, consists of minimizing plasma triglyceride levels, ideally below 500 mg/dL. Due to the reduced ability to metabolize chylomicrons, resulting from decreased lipoprotein lipase (LPL) functionality, standard pharmacological treatments such as fibrates, omega-3 fatty acids, or statins are ineffective in normalizing triglycerides (17).

### 3.1 Nutritional treatment of FCS

The baseline clinical and nutritional characteristics are summarized in Table 1. Dietary strategies include the following (18, 19): (a) to limit fat intake: 10–15% of the total caloric value (TCV) resulting in < 20–30 grams of fat/day for intakes of 2000 kcal/day, (b) to restrict the consumption of carbohydrates (<60% TCV), especially sugars and refined starches, (c) to consume purified medium-chain triglycerides (not transported by chylomicrons) to meet energy requirements, (d) to provide essential fatty acids (linoleic and *α*-linolenic; 2–4% TCV), e) to supplement fat-soluble vitamins (A, D, E, K) and micronutrients, if required, f) to avoid alcohol intake. Emphasis should be placed on the personalized application of the diet by nutritionists, providing guidance on the counting of grams of fat in foods (20). To increase adherence, culinary strategies such as buying fresh products instead of ultra-processed ones are recommended, increasing legume consumption, eliminating visible fat from the food, using alternative cooking methods (air fryers, papillote), and using spices to improve palatability (9). Unfortunately, very low-fat diets are difficult to follow, and typically have low adherence that negatively impacts long-term outcomes.

**Table 1:** Dietary modifications in familial chylomicronemia syndrome and culinary strategies to improve adherence to a very low-fat diet.

Food groups to prefer	Culinary strategies
Legumes (except soybean and peanut)	Incorporate as a main course or as a salad. Other options include: low-fat hummus, legumes puree or legume-based patties/burgers.
Wholegrain cereals	Enhance flavor with spices: paprika, pepper, curry, oregano, dill, rosemary, or thyme. Additional options include low-sodium soy sauce and monosodium glutamate (MSG).
Low-fat meats	Avoid the use of added oils (e.g., deep frying). Prefer alternate cooking methods: boiling, oven, papillote, air-frying or wrapping in aluminum foil.
Fresh fruits and vegetables	Combine a variety of colors and flavors to increase palatability and visual appeal.
Dips and sauces	Use medium-chain triglyceride (MCT) oil instead of regular oils, as tolerated.

### 3.2 Pharmacological treatment of FCS

Volanesorsen is an antisense oligonucleotide drug targeting the mRNA of APO C-III, thereby promoting TG clearance through LPL-independent pathways (12). Two phase III studies have been published to evaluate volanesorsen in severe hypertriglyceridemia. The APPROACH study (21) was a randomized, double-blind, placebo-controlled clinical trial, lasting 52 weeks, conducted in 66 patients with familial chylomicronemia. This study evaluated the administration of volanesorsen (285 mg) weekly by subcutaneous injection, with 33 patients receiving volanesorsen and 33 receiving a placebo. The results showed that volanesorsen induced an 84% reduction in circulating ApoC-III levels, translating into a 77% decrease in plasma TGL levels. Among the adverse effects, 61% of patients experienced mild to moderate discomfort at the subcutaneous injection site, and 33% had decreased platelet count. The COMPASS trial (22) was a randomized, phase 3 study that compared volanesorsen therapy versus placebo in 38 patients with severe multifactorial hypertriglyceridemia or familial chylomicronemia syndrome for 26 weeks. The volanesorsen arm achieved reductions in mean plasma triglyceride concentrations of 71.2% compared to 0.9% in the placebo group. Of note, 13% of patients receiving volanesorsen developed thrombocytopenia as an adverse effect, although only one patient had a platelet count <50,000 /μL.

Phase 3 studies showed an increase in plasma LDL cholesterol levels; however, a 27–45% decrease in plasma non-HDL cholesterol levels was observed. Importantly, the volanesorsen-treated groups experienced a reduction in acute pancreatitis events compared to the placebo group. In 2019, the European Agency EMA approved the use of volanesorsen (Waylivra 285 mg(R) in adult patients with genetically confirmed FCS who are at high risk of pancreatitis and with no adequate response to diet or triglyceride-lowering therapy.

## 4 Response to treatment and recapitulation

Dietary management remains essential in FCS but is often insufficient. In this context, volanesorsen demonstrated efficacy in reducing triglyceride levels and preventing acute pancreatitis, though side effects like thrombocytopenia warrant close monitoring. In Chile, few cases of patients diagnosed with FCS have been reported, with no reports of specific pharmacological treatments for this disease. In our country, the care of patients with rare or difficult-to-treat diseases is hindered due to the lack of a clear regulatory framework that addresses these specific needs. Only some high-cost pharmacological treatments and technologies are funded by the Chilean public health system, according to the “Ricarte Soto Law” (23, 24), which aims to guarantee access to medications and therapies for high-cost diseases.

This report has several strengths. The comparison of two sisters with familial chylomicronemia syndrome sharing the same homozygous APOA5 mutation, similar environmental exposure, and comparable disease severity provides a unique real-world framework to illustrate the clinical impact of ApoC-III inhibition. The marked divergence in clinical outcomes following treatment with volanesorsen reinforces its therapeutic efficacy beyond controlled clinical trials and highlights its relevance in routine clinical practice. In addition, this article reports the first documented case of a patient with FCS treated with volanesorsen in Chile, contributing novel regional data to the existing literature.

Several limitations should also be acknowledged. The retrospective nature of the report and the small number of cases limit the generalizability of the findings. Furthermore, the absence of standardized quality-of-life instruments and synchronized follow-up assessments restricts formal quantitative comparisons between patients. Importantly, these cases underscore broader ethical and public health challenges, as differences in clinical outcomes were not driven by biological factors but by systemic barriers to access high-cost orphan drugs. In low- to middle-income health systems, such constraints can directly influence patient outcomes, even among individuals with identical genetic conditions. Despite their shared diagnosis and similar clinical manifestations, access to treatment was completely different for the two sisters affected by FCS. The first patient achieved a significant reduction in triglyceride levels after receiving treatment with volanesorsen through a favorable Supreme Court ruling, from critical values (>6,000 mg/dL) to near-normal ranges. Unfortunately, the request for volanesorsen treatment made by patient 2, her sister, who also presented comparable clinical severity and complications, was rejected by the Chilean Supreme Court, leaving this patient without access to this innovative and effective therapy. This intra-familial contrast highlights how health system constraints and reliance on legal intervention can shape clinical outcomes in rare diseases, underscoring the urgent need for inclusive policies that align therapeutic innovation with health policy to ensure equitable access to essential treatments.

## Data Availability

The datasets presented in this article are not readily available because of ethical and privacy restrictions. Requests to access the datasets should be directed to the corresponding author.
